# SaludABLEOmaha: Improving Readiness to Address Obesity Through Healthy Lifestyle in a Midwestern Latino Community, 2011–2013

**DOI:** 10.5888/pcd12.140328

**Published:** 2015-02-12

**Authors:** Leah Frerichs, Jeri Brittin, Regina Robbins, Sharalyn Steenson, Catherine Stewart, Christopher Fisher, Terry T-K Huang

**Affiliations:** Author Affiliations: Leah Frerichs, Jeri Brittin, Regina Robbins, Sharalyn Steenson, Christopher Fisher, College of Public Health, University of Nebraska Medical Center, Omaha, Nebraska; Catherine Stewart, CommsEvolution, Ltd, London, United Kingdom.

## Abstract

**Background:**

A community’s readiness for change is a precursor to the effective application of evidence-based practices for health promotion. Research is lacking regarding potential strategies to improve readiness to address obesity-related health issues in underserved communities.

**Community Context:**

This case study describes SaludABLEOmaha, an initiative to increase readiness of residents in a Midwestern Latino community to address obesity and adopt healthy lifestyles.

**Methods:**

SaludABLEOmaha emphasized 2 core approaches, youth activism and collaboration among public and private institutions, which we applied to planning and implementing tactics in support of 3 interconnected strategies: 1) social marketing and social media, 2) service learning in schools (ie, curricula that integrate hands-on community service with instruction and reflection), and 3) community and business engagement. Following the Community Readiness Model protocol (http://triethniccenter.colostate.edu/communityReadiness.htm), structured interviews were conducted with community leaders and analyzed before and 2.5 years after launch of the program.

**Outcome:**

The community increased in readiness from stage 3 of the Community Readiness Model, “vague awareness,” at baseline to stage 5, “preparation,” at follow-up.

**Interpretation:**

SaludABLEOmaha improved community readiness (eg, community knowledge, community climate), which probably contributed to the observed increase in readiness to address obesity through healthy lifestyle. Community mobilization approaches such as youth activism integrated with social marketing and social media tactics can improve community responsiveness to obesity prevention and diminish health disparities.

## Background

National trends of obesity among children aged 2 to 19 years in the United States may be leveling (1), yet childhood obesity among Latinos continues to increase (2). In the past decade, major metropolitan areas addressed childhood obesity with policy and environmental interventions; however, reductions among Latino children are not as great as those among non-Latino white children (3). Research is lacking regarding strategies to increase readiness to address obesity in underserved communities.

Community readiness, a theory- and practice-based construct, is the degree to which a community is ready to take action on an issue (4). Research suggests that a community has to be ready to address a given issue before evidence-based practices for health promotion can be introduced into and adopted by the community (5). The Community Readiness Model (CRM) (http://triethniccenter.colostate.edu/communityReadiness.htm) defines 9 stages of community readiness (Box) and serves as a tool to help communities mobilize for change. CRM has been shown to help underserved communities, including Latino populations, address many health issues (4,6).

**Box. Description of Stages in the Community Readiness Model**

**Table:** 

Community Readiness Model Stages	Definition
Stage 1. No awareness	Issue is not generally recognized by the community or community leaders as a problem (or it may not be an issue).
Stage 2. Denial or resistance	At least some community members recognize that the issue is a concern, but there is little recognition that it might be occurring locally.
Stage 3. Vague awareness	Most feel that there is a local concern, but there is no immediate motivation to do anything about it.
Stage 4. Preplanning	There is clear recognition that something must be done, and there may even be a group addressing the issue. However, efforts are not focused or detailed.
Stage 5. Preparation	Leaders begin planning in earnest. Community offers modest support of efforts.
Stage 6. Initiation	Enough information is available to justify efforts. Activities are under way.
Stage 7. Stabilization	Activities are supported by administrators or community decision makers. Staff are trained and experienced.
Stage 8. Confirmation or expansion	Efforts are in place. Community members feel comfortable using services, and they support expansions. Local data are regularly obtained.
Stage 9. High level of community ownership	Detailed and sophisticated knowledge exists about prevalence, causes, and consequences. Effective evaluation guides new directions. Model is applied to other issues.

Two studies described using CRM to identify appropriate first steps in addressing childhood obesity in non-Latino communities (7,8). One of those studies used awareness-raising strategies (eg, health fairs, newspaper articles) in a rural community at the lowest stage of readiness (7). These studies, however, did not systematically assess the effect of these strategies on readiness levels. We used CRM to design and prospectively evaluate an initiative in an underserved Latino community to increase community readiness. The initiative used 2 approaches, youth advocacy and cross-sectoral (ie, collaboration among public and private institutions) to increase sustainability and community involvement (9,10) in implementing dimensions of community readiness.

## Community Context

From 2000 to 2010, the Latino population of the Omaha, Nebraska, metropolitan area increased 87%. In 2013 Latinos made up 12% of the total population (11). Our initiative was conducted in South Omaha where 51% of all Omaha Latinos live. Depending on zip code, Latinos make up from 30% to 55% of the area’s total population (11).

One-fifth of all Omaha Latino families and 37.7% of Latino single-parent families with a Latino mother have incomes at or below the federal poverty level (11). Among Latino adults aged 25 years or older, 47% have less than a high school education compared with 5.4% of non-Latino whites. A telephone survey of a random sample of Omaha households found that 31% of youths aged 12 to 19 years in South Omaha were overweight or obese, compared with 20% in northwest and southwest Omaha where residents are primarily non-Latino white (12).

One of the greatest assets of the South Omaha Latino community is the South Omaha Community Care Council (SOCCC), a coalition with a partnership base of over 150 organizations. During the past several years, this coalition has enhanced collaboration and communication among area universities and health and social service agencies. Several major institutions that address health issues have also been established in the community. For example, a federally qualified community health center was built in 2009; it provides a range of preventive health care and treatment services and has a fitness and recreation facility of over 120,000 sq ft. At the outset of the SaludABLEOmaha initiative, we found that few activities targeted childhood obesity and that those that did were not coordinated across the community (9).

In 2011, we partnered with the South Omaha Latino community (eg, SOCCC and its coalition partners) to develop SaludABLEOmaha, an initiative whose main objective was to increase the community’s readiness to address obesity prevention, particularly among youths. Our objective was to build a sustainable initiative with potential to increase the community’s engagement in preventing obesity and diabetes and recognition of how these conditions relate to healthy eating and physical activity.

## Methods

### Community Readiness Model assessments

CRM uses a structured interview to measure 6 dimensions of community readiness to deal with any public health issue: presence of related community efforts, community’s knowledge about these efforts, leadership support for dealing with the issue, community climate (ie, attitude of community leaders toward the issue), community’s knowledge about the issue, and availability of resources to implement a program to deal with the issue (Table) (6). The interview is reliable (reported scorer ratings agree 92% of the time) and well-validated across topics (eg, substance use, cancer) and populations (4,6). To achieve an accurate representation, CRM requires 4 to 6 interviews with people knowledgeable about the community and from sectors (eg, government institutions, private and community-based organizations) relevant to the issue (4,6). We conducted 10 interviews at baseline from February through May 2011 and 7 interviews 2.5 years post-baseline, from October through December 2013.

**Table:** SaludABLEOmaha’s Strategies and Tactics Used to Address Low Levels of Community Readiness by Dimension, Omaha, Nebraska, 2011–2013

Community Readiness Model Dimensions (Definition)	SaludABLEOmaha Strategy	Tactics to Address Low Readiness Levels
***Community knowledge of the issue.*** (To what extent do community members know about the causes of the problem, consequences, and how it affects their community?)	Social marketing and social media	• Webisode series introduced the issue of obesity and related lifestyle behaviors with emotionally appealing storylines (eg, a storyline involves a character that discusses her concern for a friend’s eating and lifestyle because she has a parent who has diabetes). • Website with multimedia and visual materials to introduce basic obesity and healthy lifestyle information. • Posts on social media that include infographics about healthy eating.
***Community Climate.*** (What is the prevailing attitude of the community toward the issue? Is it one of helplessness or one of responsibility and empowerment?)		

***Leadership*** (To what extent are appointed leaders and influential community members supportive of the issue?)	Service learning in schools	• Youth activists engaged school leadership to garner support for changes to the school food environment. • Youth activists implemented a low-resource and trial-able change to the school environment (eg, a color-coded labeling campaign in the school cafeteria for 9 weeks).
***Community efforts*** (To what extent are there efforts, programs, and policies that address the issue?)		

***Community knowledge of efforts*** (To what extent do community members know about local efforts and their effectiveness, and are the efforts accessible to all segments of the community?)	Community and business engagement	• Youth activists set up an information booth at the annual Cinco de Mayo event to increase awareness in the community about their efforts • Youth activists encouraged community members to register online as SaludABLEOmaha supporters, indicating their support for growing resources to address obesity as a first step to building support for the initiative.
***Resources*** (To what extent are local resources — people, time, money, space, etc. — available to support efforts?)		

At baseline, initiative partners helped develop a list of key respondents from organizations and institutions with a stake in addressing obesity prevention. We recruited 10 interviewees from schools, medical professions, social service organizations, and recreational facilities. All persons interviewed at baseline were approached for follow-up interviews 2.5 years later; however, 4 were no longer in their previous position, 2 were unresponsive to interview requests, and 1 declined participation. To collect adequate data, we recruited people who replaced, or were in similar position to, those interviewed at baseline. A total of 7 people participated in follow-up interviews, 3 from the baseline sample and 4 replacements.

Interviews were conducted in person and were audio-recorded by trained research team members. Interviews ranged from 20 to 45 minutes. Two interviews at baseline and 1 interview at follow-up were conducted in Spanish and the remainder were conducted in English. We transcribed audio recordings verbatim. Two trained evaluators scored the interview transcripts independently. For each person interviewed, readiness was measured for each of the 6 CRM dimensions on dimension-specific anchored scales (4,6). The anchored scales ranged in whole numbers from 1 to 9 for each dimension; 9 is the most favorable score. The evaluators compared and discussed independent scoring to reach consensus on final scores. To compute the total CRM score for each respondent, the ratings of all 6 dimensions were averaged. The overall stage of readiness at baseline and follow-up was determined by rounding the average score to the lowest calculated CRM stage (Box). The study was approved by the institutional review board of the University of Nebraska Medical Center.

### Theoretical frameworks and cross-cutting approaches

**Figure 1:**
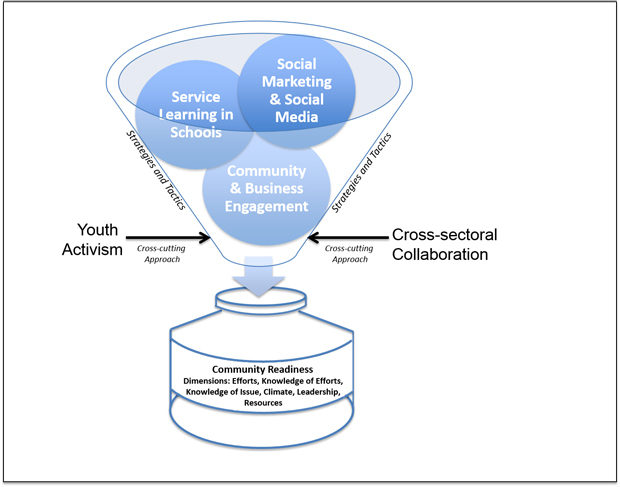
SaludABLEOmaha implementation framework. The framework includes 2 cross-cutting approaches (youth activism and cross-sectoral collaboration) and 3 interconnected strategies (service learning in schools, social marketing and social media, and community and business engagement). These approaches were designed to increase community readiness, which includes dimensions of efforts, knowledge of efforts, knowledge of issue, climate, leadership, and resources.

The community’s stage of readiness was low at baseline, and initial phases of SaludABLEOmaha focused on establishing relationships and building partnerships, which created the necessary infrastructure for youth advocacy and cross-sectoral collaboration (eg, a partnership with the community’s public high school created an avenue for recruiting youth activists) (9). We used this infrastructure to implement tactics within 3 interconnected core strategies: 1) social marketing and social media, 2) service learning in schools (ie, curricula that integrate hands-on community service with instruction and reflection), and 3) community and business engagement (Figure 1). The tactics were designed to target dimensions of community readiness and were tailored for the community’s low stage of readiness (Table).

**Figure 2:**
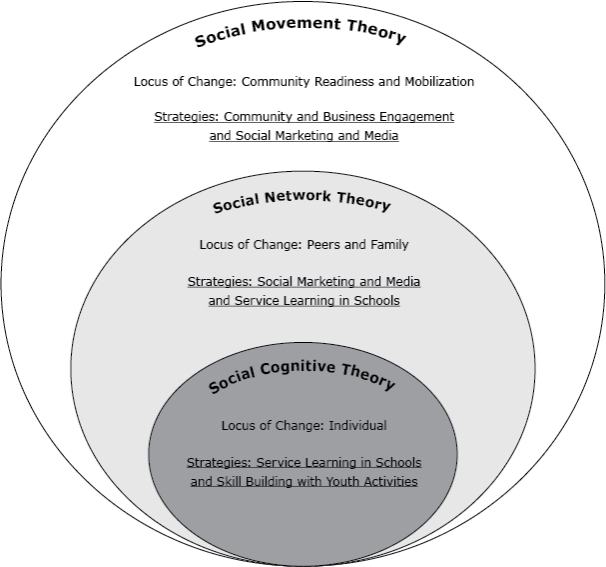
SaludABLEOmaha’s 3 theoretical frameworks. SaludABLEOmaha draws on each theory for specific strategies and loci of change.

The initiative was built on the integration of social cognitive theory (13), social network theory (14), and social movement theory (15) (Figure 2). We used social cognitive theory, which is described in detail elsewhere (9), to focus on advocacy skill-building among the youth activists (ie, observational and experiential learning techniques to increase youth self-efficacy to address community health). Social network theory was the basis for using social marketing and social media strategies to spread messages through youth activist peer and community partner networks. Finally, in accordance with social movement theory, we used community and business engagement combined with social marketing and media strategies to align the demand for health (ie, improving norms of healthy lifestyles) and the supply of health contexts (ie, improving policy and environmental supports for healthy lifestyles).

Partnerships among public and private institutions were developed throughout the initiative. For example, the South Omaha community’s public high school was a key partner that provided infrastructure to sustain youth activism. High school administrators and staff assisted with recruiting and training 10 to 20 youth activists per year. SaludABLEOmaha’s social marketing and social media strategies included private partners, such as a college undergraduate-run public relations firm. Media and communication agencies provided in-kind time and expertise to mentor youth activists and assisted with designing SaludABLEOmaha’s marketing platforms (eg, website) and materials.

Youth activists were the heart of SaludABLEOmaha; they provided important links to the South Omaha community. We recruited and trained cohorts of 6 to18 youths from Omaha South High School each year. The first cohort spent an intensive summer session developing the initiative’s brand and logo (9). This was a critical first step to creating community-relevant and culturally appropriate frameworks and messaging strategies. Each cohort led development and implementation of tactics to increase community readiness within the 3 interconnected core strategies (Table).

### Interconnected strategies targeting community readiness dimensions

**Social marketing and social media.** CRM dimensions, “community knowledge of the issue” and “community climate,” scored lowest at baseline and were a focus of SaludABLEOmaha. The first youth activist cohort developed the SaludABLEOmaha brand and logo to be relevant to the community and to serve as a platform to initiate communication and raise awareness around health. “Saludable” in Spanish means “healthful” and “ABLE” stands for “Attitude/Actitud,” “Balance/Balance,” “Leadership/Liderazgo,” and “Energy/Energía,” which provides a springboard for communication around a core message (ie, “With these qualities we can make healthy choices easier and cheaper for our families and community.”).

Targeted communication campaigns were directed to Latino youths and their parents and used storytelling and visual media to increase knowledge and shift norms about obesity in relation to healthy lifestyles. We produced a series of short videos and disseminated them through social media platforms (Web page: http://saludableomaha.org/, Facebook: https://www.facebook.com/SaludableOmaha, and YouTube: https://www.youtube.com/channel/UCQBY6hEtLQgQDIh1vtl4bQQ). Videos targeting youths included messages that emphasized the social benefits of healthy lifestyles. For example, a “webisode” series followed a romance between a young woman who highly valued healthy eating and a young man who tried to impress her with his cooking skills. Videos targeting parents had a stronger emphasis on messages related to the nutrition-related benefits of healthy foods with consideration of community norms and values. For example, several videos featured dieticians demonstrating simple recipes and snacks and showcased the increased nutritional value —and greater volume — of healthy foods compared with junk foods popular in the community.

The SaludABLEOmaha brand is also integrated into specific initiative strategies and is disseminated through in-person settings. For example, youth activists implemented a school cafeteria labeling and marketing campaign, which was co-branded by SaludABLEOmaha and other project partners (ie, Omaha Public School’s Nutrition Services and Live Well Omaha Kids). Finally, we distributed promotional materials with the SaludABLEOmaha brand and logo (eg, water bottles, stickers) at in-person events (eg, community gatherings such as Cinco de Mayo celebrations) to increase brand awareness.

**Service learning in schools.** This strategy primarily targeted CRM dimensions of leadership and community efforts. With assistance from researchers, teachers, and leaders of community health organizations, youth activists engaged with school leaders to advocate for interventions in the school setting that required minimal resources and were amenable to trial. Youths conducted audits of their school’s health-related environment, developed strategic plans to address identified concerns, and met with school administrators to discuss potential activities. From 2011 through 2013, youths piloted a 9-week color-coded-labeling campaign in the school cafeteria and a peer-to-peer marketing campaign to promote healthy snack and drink choices.

**Community and business engagement.** This strategy primarily targeted community knowledge of SaludABLEOmaha efforts and resources. In addition to online dissemination, SaludABLEOmaha used on-the-ground activities to spread awareness in the community about the initiative’s efforts. A first step was to build relationships with major community stakeholders in public and private sectors (eg, schools, social service agencies, churches, grocery stores). For example, we attended SOCCC meetings to update attendees on activities and to network with other organizations. Also, from 2011 through 2013, youth activists attended 5 major community gatherings (eg, Cinco de Mayo events) and 4 community coalition meetings to personally engage with fellow community members.

## Outcome

**Figure 3:**
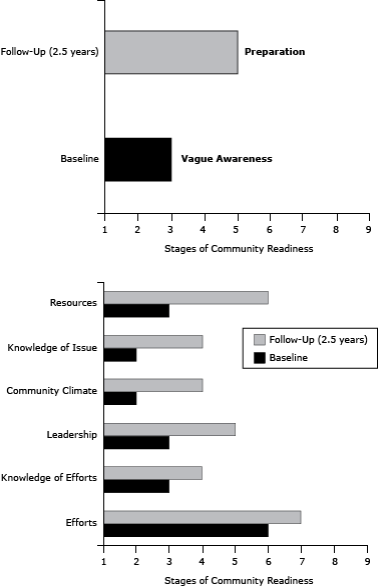
South Omaha Latino community’s stage of readiness to address childhood obesity and anchored community readiness rating scores by readiness dimension, at baseline in 2011 and 2.5 year follow-up in 2013. **Table:** 

From baseline to follow-up, the overall community’s readiness to address childhood obesity increased (Figure 3). At baseline, the community was at stage 3, “vague awareness,” indicating that most community members felt that childhood obesity was a concern but had no immediate motivation to address it. After 2.5 years, SaludABLEOmaha had implemented many tactics that targeted improvements among the dimensions of community readiness; the community had progressed to stage 5, “preparation,” indicating that leaders had begun planning efforts to address childhood obesity and that the community offered modest support. Each of the 6 community readiness dimensions’ anchored rating scores increased (Figure 3). The “efforts” dimension was relatively high at baseline (anchored rating of 6) and increased by 1. The anchored rating scores for community “knowledge of the issue” and “community climate” were low at baseline, and each increased from a score of 2 to 4. The dimension of “resources” had the largest absolute increase — from 3 to 6 on the anchored rating scale. Finally, the “leadership” dimension score increased from 2 to 4 and the “community knowledge of efforts” dimension score increased from 3 to 4.

Community Readiness Dimensions	Baseline	Follow-Up (2.5 years)
Overall community readiness score	3	5
Efforts	6	7
Knowledge of Efforts	3	4
Leadership	3	5
Community Climate	2	4
Knowledge of Issue	2	4
Resources	3	6

The combination of cross-sectoral collaboration and youth activism was key to achieving community relevance and reach. For example, our public school partner, Omaha South High School, was critical to recruiting youth activists for the initiative. In addition, private media partners worked with youths to co-create social marketing materials such as an original music video, “Dedication.” “Dedication” is SaludABLEOmaha’s Facebook post with the highest unpaid reach (over 20,000 users from Omaha).

## Interpretation

SaludABLEOmaha’s goal was to increase community readiness. We found that 2.5 years after the initiative’s launch, the South Omaha Latino community had gone from a stage of no immediate motivation to address childhood obesity to a stage of community support and planning to address obesity. The core approaches, youth activism and collaboration of public and private institutions, were key to the growth and sustainability of SaludABLEOmaha and central to the development of interconnected strategies that were tailored to increase dimensions of community readiness.

SaludABLEOmaha probably played a role in increasing community readiness. SaludABLEOmaha’s social marketing and social media tactics focused on shifting the South Omaha Latino community’s knowledge, values, and norms around obesity, which were relevant to dimensions of community “knowledge of the issue” and “climate.” During the interviews, 4 of the 7 participants specifically referenced SaludABLEOmaha and made statements such as, “I think people are more conscious about [obesity] . . . they look at [SaludABLEOmaha’s] website.” At the same time, other organized community efforts have also contributed to increased community readiness. For example, the increased score of the “leadership” dimension was probably influenced by SOCCC activities. Four of 7 interview participants noted the Council’s leadership in the community over the past 2 years, making statements such as, “I know that [obesity is] at the forefront of [SOCCC’s] . . . agenda.”

Recruiting and maintaining active youth involvement can be a challenge for an initiative like SaludABLEOmaha. In collaboration with the Omaha South High School, we explored different recruitment strategies (eg, school-wide announcements, recruitment through art programs, partnership with student clubs), leading to variation in the total number of youths involved and their level of engagement each year. This approach led to recruitment of youths with various talents and created the interest and capacity to implement a range of activities. The most recent youth-activist cohort was trained on a teen-mentoring nutrition program, which they will implement with middle school students in the 2014–2015 school year.

Partnerships among public and private institutions are also important to community engagement. Experts recommend that obesity prevention efforts include multiple sectors to build human, financial, and regulatory capacity for change (10). SaludABLEOmaha partnerships were formed with recognition that public and private sectors have different goals and included activities that would be mutually beneficial. For example, a new community outreach strategy is under way that will further develop relationships with local South Omaha restaurants whose primary goals by nature are geared toward revenue generation and customer satisfaction. Youth activists are in the process of recruiting restaurants to display a SaludABLEOmaha table centerpiece that contains tips to improve healthy eating choices in the restaurant. As a credible endorsement of the project, the county health department will co-brand the display, and SaludABLEOmaha will use its relationships with local media to provide free promotion for participating restaurants.

SaludABLEOmaha included an emphasis on branding. There is an emerging shift to consider how social marketing can go beyond addressing individual behavior change and address the social values and experiences in which behavior is embedded (16). Brands are noted for their ability to embody multiple behavior change messages and position these messages with the social and physical environment of targeted audiences (17). The SaludABLEOmaha brand was co-created with local youths to embody the complex set of obesity-related behaviors and to position healthy lifestyles as socially desirable within the South Omaha Latino community. This may be an effective future approach for communities with low readiness levels.

SaludABLEOmaha marketing campaigns used social media. Marketing through social media is cost-effective and can reach large audiences (18). Additionally, social media have transformed communication and provided opportunities for enhanced interaction (19). However, we found that considerable effort was needed to keep pace with target audience migration across social media platforms. For example, our founding youth activist cohort primarily used Facebook, but youths recently migrated to Twitter and Instagram. Each platform has different features and services, which require significant adaption of messaging strategies.

Our study had several limitations. The researchers who conducted CRM interviews were often involved with SaludABLEOmaha program activities. Respondents possibly exaggerated responses because of social desirability bias; however, CRM provides a structured interview tool and scoring process that lessens this concern. Also, we did not have information on community readiness from comparison communities. Thus, although the increase in community readiness is promising, we cannot claim that SaludABLEOmaha is the sole cause.

Our findings indicated an increase in the South Omaha Latino community’s readiness to address obesity-related health issues. Future efforts need to be multilevel and cross-sectoral, and they should include community mobilization approaches, such as youth activism integrated with social marketing and social media. Such efforts can play a role in improving community readiness and can, in coordination with additional initiatives (eg, interventions within the health care setting, outreach to parents through faith-based organizations), improve responsiveness to obesity interventions and diminish health disparities in underserved communities.
